# A smoothing‐based goodness‐of‐fit test of covariance for functional data

**DOI:** 10.1111/biom.13005

**Published:** 2019-04-06

**Authors:** Stephanie T. Chen, Luo Xiao, Ana‐Maria Staicu

**Affiliations:** ^1^ Department of Statistics North Carolina State University Raleigh North Carolina

**Keywords:** functional data analysis, Functional principal components analysis, hypothesis testing, linear mixed effects models, longitudinal data analysis

## Abstract

Functional data methods are often applied to longitudinal data as they provide a more flexible way to capture dependence across repeated observations. However, there is no formal testing procedure to determine if functional methods are actually necessary. We propose a goodness‐of‐fit test for comparing parametric covariance functions against general nonparametric alternatives for both irregularly observed longitudinal data and densely observed functional data. We consider a smoothing‐based test statistic and approximate its null distribution using a bootstrap procedure. We focus on testing a quadratic polynomial covariance induced by a linear mixed effects model and the method can be used to test any smooth parametric covariance function. Performance and versatility of the proposed test is illustrated through a simulation study and three data applications.

## 1 Introduction

Functional data have become increasingly common in fields such as medicine, agriculture, and economics. Functional data usually consist of high frequency observations collected at regular intervals, see Ramsay and Silverman (2002, 2005) for an overview of methods and applications. By comparison, longitudinal data typically consist of repeated observations collected at a few time points varying across subjects. In recent years, functional data methods have been successfully extended and applied to longitudinal data (James et al., 2000; Yao et al., 2005). While these methods are more flexible, their estimation and interpretation are more cumbersome than longitudinal methods and require more sampling units or observations for accurate and reliable estimates. Thus, it is natural to question if such flexibility is truly necessary. This article focuses on comparing longitudinal data methods with functional data methods. For example, we consider the case of testing if a simple linear mixed effects model is sufficient for longitudinal data or if a more complex functional data model is required.

This work is motivated by the CD4 cell count dataset from the Multicenter AIDS Cohort Study (Kaslow et al., 1987). CD4 count is a key indicator for AIDS disease progression, and understanding its behavior over time is critical for monitoring HIV+ patients. The dataset is highly sparse, with 5 to 11 irregularly‐spaced observations per subject. CD4 counts have been extensively analyzed using longitudinal data methods, e.g., semiparametric and linear random effects models (Taylor et al., 1994; Zeger and Diggle, 1994; Fan and Zhang, 2000). Recently, functional data methods have also been applied to this data (Yao et al., 2005; Goldsmith et al., 2013; Xiao et al., 2018). While the nonparametric functional data methods are highly flexible and better adapt to subject‐specific patterns, they are more difficult to implement and interpret compared to the parametric approaches. Therefore it is of interest to test whether the simpler longitudinal methods are sufficient for the data. To the authors’ best knowledge, no formal testing procedure exists for this application.

The inherent difference between functional and traditional longitudinal data methods is in the correlation model between repeated observations. For functional methods, the covariance within a subject is assumed to be smooth with an unknown nonparametric form. The covariance can be estimated by smoothing the sample covariance (Besse and Ramsay, 1986; Yao et al., 2005; Xiao et al., 2018) or constructing a reduced rank approximation by estimating basis functions from smoothed sample curves (James et al., 2000; Peng and Paul, 2009). In contrast, longitudinal data approaches typically assume a simple parametric covariance structure with a few parameters, such as autoregressive or exponential (see Diggle et al. (2002) for an overview), or induced by a random effects model (Laird and Ware, 1982).

Existing work on testing parametric versus nonparametric functions is limited to density and regression functions for the response variable, but has been extended to settings such as semiparametric and functional models; see González‐Manteiga and Crujeiras (2013) for a recent review. Hardle and Mammen (1993) propose a smoothing‐based goodness‐of‐fit statistic for regression functions, derive the asymptotic normal distribution, and develop a “wild” bootstrap algorithm for finite samples. Comparisons have also been applied to functional regression for model diagnostics and evaluating assumptions (Chiou and Muller, 2007; Bucher et al., 2011) and testing functional coefficients (Swihart et al., 2014; McLean et al., 2015; Kong et al., 2016). The proposed method is an extension of smoothing‐based methods to test the form of the covariance function.

For high‐dimensional multivariate data, where observation points are regular and balanced (same for all subjects), a number of methods exist to test an identity or spherical covariance matrix against an unstructured alternative (Ledoit and Wolf, 2002; Bai et al., 2009). Recently, Zhong et al. (2017) developed a general goodness‐of‐fit test that can be applied to many common parametric covariances. However, these methods are ill‐suited for the comparison between functional and longitudinal data models because they (a) fail to account for the underlying smoothness of the process and (b) require data observed at fixed time points for all subjects, i.e., a *(fixed) common design* The CD4 dataset has an irregular design where time points differ for each subject, so cannot be tested with these approaches. Note that the *random design*, where observed time points are independent between and within the subjects, is a special case of the irregular design. Common or random designs are typically assumed in theoretical studies of functional data (Cai and Yuan, 2011).

The objective of this article is to develop a testing procedure for comparing parametric longitudinal versus nonparametric functional data covariance models applied to repeated measured data with irregular and/or highly frequent sampling design. Note that longitudinal data with only a few repeated measurements per subject with a regular sampling design is not within the scope of this article. Selecting an adequate covariance model is critical, because model misspecification can bias estimation and inference, while an unnecessarily complex model can slow computation and interfere with model interpretation. We propose a goodness‐of‐fit test based on the difference between the estimated parametric and nonparametric covariances, inspired by Hardle and Mammen (1993). Compared to Zhong et al. (2017) for high‐dimensional multivariate data, our test statistic can be evaluated using a more flexible modeling approach that accounts for general designs and exploits the underlying smoothness of repeated observations. However, deriving the distribution of the test statistic is challenging and we use bootstrapping to approximate the null distribution. To demonstrate performance and versatility of the proposed test, we present a simulation study and three data applications.

The remainder of this article is organized as follows. Section 2 presents the statistical model and hypothesis test, Section 3 details the proposed test, and Section 4 describes our implementation. Section 5 outlines extensions to general smooth covariance functions. Section 6 presents a simulation study. Section 7 details three applications to diffusion tensor imaging, child growth, and CD4 cell count. Finally, Section 8 summarizes the article and discusses limitations of the proposed test.

## 2 Statistical Framework

Consider functional or longitudinal data $\left\{(t_{\mathrm{ij}},Y_{\mathrm{ij}})\in T\times \mathbb{R}:i=1,\ldots ,n,j=1,\ldots ,m_{i}\right\}$ where *i* denotes the subject index, *j* denotes the visit index, and $Y_{\mathrm{ij}}$ is the measurement for the *i*‐th subject at time $t_{\mathrm{ij}}$. Here, *n* is the number of subjects and $m_{i}$ the number of observations for the *i*‐th subject, which can vary across subjects. Assume that T=[a,b] is a closed and compact domain. Data are often observed with noise, so we posit the model

(1) $$Y_{\mathrm{ij}}=\mu (t_{\mathrm{ij}})+X_{i}(t_{\mathrm{ij}})+\varepsilon_{\mathrm{ij}}.$$

Here μ(t) is a smooth mean function, $X_{i}$ is a zero‐mean Gaussian random function independent between subjects, and $\varepsilon_{\mathrm{ij}}$ is Gaussian white noise independently and identically distributed with zero mean and variance $\sigma^{2}$, independent of $X_{i}$.

Let $G(t,t^{\prime })=\mathrm{Cov}\{X_{i}(t),X_{i}(t^{\prime })\}$ be the covariance function of $X_{i}$. Assume that G is a smooth, positive semidefinite bivariate function defined on $T^{2}$.

We are interested in the form of the covariance, and would like to test the hypothesis that G has a known parametric form against a general alternative. Motivated by the CD4 dataset, which has previously been fit with a linear random intercept and slope model, we focus on the quadratic polynomial function

(2) $$G_{0}(t,t^{\prime })=\sigma_{0}^{2}+\sigma_{01}(t+t^{\prime })+\sigma_{1}^{2}{\mathrm{tt}}^{\prime },$$

where ($\sigma_{0}^{2},\sigma_{01},\sigma_{1}^{2}$) are unknown parameters. Because this covariance is induced by the linear random effects model $X_{i}(t)=b_{0i}+b_{1i}t$, where $b_{i}=(b_{0i},b_{1i}{\;)}^{T}$ are random effects with zero mean and $\mathrm{Var}(b_{i0})=\sigma_{0}^{2}$, $\mathrm{Var}(b_{i1})=\sigma_{1}^{2}$, and $\mathrm{Cov}(b_{i0},b_{i1})=\sigma_{01}$, testing $G_{0}$ is equivalent to testing if a linear random (or mixed) effects model is sufficient for the data. Note that this is a specific case of the general linear random effects model $X_{i}(t)=\sum_{k=1}^{K}b_{\mathrm{ik}}\varphi_{k}(t)$ for random effects $b_{\mathrm{ik}}$ with zero mean and variance $\sigma_{k}^{2}$ and known functions $\varphi_{k}(t)$, which has covariance function $G_{0}(t,t^{\prime })=\sum_{k=1}^{K}\sigma_{k}^{2}\varphi_{k}(t)\varphi_{k}(t^{\prime })+2\sum_{k<k^{\prime }}\sigma_{{\mathrm{kk}}^{\prime }}\varphi_{k}(t)\varphi_{k^{\prime }}(t^{\prime })$, where $\sigma_{{\mathrm{kk}}^{\prime }}=\mathrm{Cov}(b_{\mathrm{ik}},b_{{\mathrm{ik}}^{\prime }})$. While we focus on (2), the proposed test can be easily adapted for the more general random effects case or any smooth parametric covariance with finite parameters, as discussed in Section 5. Ideally, scientific or expert knowledge about the underlying process should guide the choice of $G_{0}$. If such information is unavailable, a commonly used and interpretable structure would be preferred.

Formally, the hypothesis test can be written as

(3) $$H_{0}:G(t,t^{\prime })=G_{0}(t,t^{\prime })\;\mathrm{versus}\;H_{A}:G(t,t^{\prime })\neq G_{0}(t,t^{\prime }).$$

Under the null hypothesis, the covariance has a specific parametric form with finite parameters. Under the alternative hypothesis, the covariance function is assumed only to be smooth and positive semidefinite. This flexibility may better capture heterogeneity across subjects but is hard to estimate and interpret compared to a parametric model. Therefore, it is desirable to test goodness‐of‐fit for these two types of models. In the following section, we propose a distance‐based goodness‐of‐fit test for (3) that can be applied to functional data with either a dense common or sparse irregular sampling design.

## 3 Smoothing‐based Test

We propose a test statistic based on the distance between the covariance functions estimated under the null and alternative hypotheses, respectively. In the remainder of this section, we describe covariance estimation under the null and alternative hypotheses, and then introduce our test statistic. The smooth mean μ(t) can be estimated non‐parametrically with spline smoothing (Ruppert et al., 2003; Wood, 2003), allowing us to consider only the de‐meaned data ${\tilde{Y}}_{\mathrm{ij}}=Y_{\mathrm{ij}}-\hat{\mu }(t_{\mathrm{ij}})$ for modeling $X_{i}(t_{\mathrm{ij}})+\varepsilon_{\mathrm{ij}}$. See Section 4 for details.

### 3.1 Null model

Under the null hypothesis, $G=G_{0}$ is a quadratic polynomial covariance, corresponding to

(4) $$\begin{array}{cc} X_{i}(t) & =b_{0i}+b_{1i}t\\ (b_{i0},b_{1i}{\;)}^{T} & ∼N\left(0,V_{0}=\left[\begin{array}{cc} \sigma_{0}^{2} & \sigma_{01}\\ \sigma_{01} & \sigma_{1}^{2} \end{array}\right]\right). \end{array}$$

Here, $X_{i}(t)$ is a linear random effects model with subject‐specific random intercepts and slopes, $b_{0i}$ and $b_{1i}$, respectively. Let ${\tilde{Y}}_{i}$ be the $m_{i}$‐length vector of de‐meaned observations for the *i*‐th subject observed at times $t_{i}=(t_{i1},\ldots ,t_{{\mathrm{im}}_{i}}{\;)}^{T}$, and $V_{i}=[1,t_{i}]V_{0}[1,t_{i}{\;]}^{T}+\sigma^{2}I_{m_{i}}$ be the corresponding covariance matrix, where 1 is a $m_{i}$‐length vector of ones and $I_{m_{i}}$ is a $m_{i}\times m_{i}$ identity matrix. Then the unknown parameters in model (4) can be estimated by maximizing the log‐likelihood $\ell (V_{0},\sigma^{2}|{\tilde{Y}}_{i},t_{i})=\sum_{i=1}^{n}-\frac{1}{2}(\text{log}|V_{i}|+{\tilde{Y}}_{i}^{T}V_{i}^{-1}{\tilde{Y}}_{i})$, where $\left|V_{i}\right|$ is the determinant of the matrix $V_{i}$, using an expectation‐maximization (EM) or Newton–Raphson algorithm, as outlined in Lindstrom and Bates (1988).

### 3.2 Alternative model

Under the alternative hypothesis, the covariance function has a smooth, nonparametric form. Approximate $G_{A}$ by smoothing the sample covariance using tensor product regression splines as $G(t,t^{\prime })=\sum_{h,\ell =1}^{H}\theta_{h\ell }B_{h}(t)B_{\ell }(t^{\prime })$, where $\left\{B_{h}(t):h=1,2,\ldots ,H\right\}$ are a sequence of cubic B‐spline basis functions defined over T and ${\hat{\theta }}_{h\ell }$ are coefficients estimated by minimizing the least squares expression

(5) $$\sum_{i=1}^{n}\sum_{1\leq j\neq j^{\prime }\leq m_{i}}{\left\{{\tilde{Y}}_{\mathrm{ij}}{\tilde{Y}}_{{\mathrm{ij}}^{\prime }}-\sum_{h,\ell =1}^{H}\theta_{h\ell }B_{h}(t_{\mathrm{ij}})B_{\ell }(t_{{\mathrm{ij}}^{\prime }})\right\}}^{2},$$

under the natural symmetry constraint that $\theta_{h\ell }=\theta_{\ell h}$. Denote the estimated alternative covariance as ${\hat{G}}_{A}(t,t^{\prime })=\sum_{h,\ell =1}^{H}{\hat{\theta }}_{h\ell }B_{h}(t)B_{\ell }(t^{\prime })$.

The measurement error, $\sigma^{2}$, in equation (1) can be estimated following Yao et al. (2005) and Goldsmith et al. (2013) by averaging the distance between the diagonals of the raw sample covariance, i.e., ${\tilde{Y}}_{\mathrm{ij}}^{2}$ for $1\leq j\leq m_{i},1\leq i\leq n$, and ${\hat{G}}_{A}$. To mitigate boundary effects, only the middle 50% of T is considered (Staniswalis and Lee, 1998; Yao et al., 2005).

### 3.3 Test statistic

Using the estimated null and alternative covariances, ${\hat{G}}_{0}$ and ${\hat{G}}_{A}$, the proposed test statistic is the Hilbert–Schmidt norm distance

(6) $$T_{n}=||{\hat{G}}_{A}-K{\hat{G}}_{0}|{|}_{\mathrm{HS}},$$

where $||f|{|}_{\mathrm{HS}}=\sqrt{\int \int f(t,t^{\prime }{\;)}^{2}\mathrm{dt}{\mathrm{dt}}^{\prime }}$ for bivariate function *f* and $K{\hat{G}}_{0}$ is the smoothed null covariance estimate using tensor‐product B‐splines. That is, replace ${\tilde{Y}}_{\mathrm{ij}}{\tilde{Y}}_{{\mathrm{ij}}^{\prime }}$ with ${\hat{G}}_{0}(t_{\mathrm{ij}},t_{{\mathrm{ij}}^{\prime }})$ in the least squares expression (5) to estimate $\theta_{0,\mathrm{hl}}=\theta_{0,\mathrm{lh}}$ so $K{\hat{G}}_{0}(t,t^{\prime })=\sum_{h,\ell =1}^{H}{\hat{\theta }}_{0,\mathrm{hl}}B_{h}(t)B_{\ell }(t^{\prime })$. Using the smoothed null eliminates the bias from nonparametric function estimation and is common practice for nonparametric regression tests; see, e.g., Hardle and Mammen (1993). A large $T_{n}$ indicates that the null parametric covariance approximates the true covariance poorly. The null distribution of $T_{n}$ is difficult to derive as estimation of the alternative is based on second moments of the observed responses. Moreover, even in settings where the null distribution of distance‐based test statistic is available, Hardle and Mammen (1993) show that the test statistic converges slowly and recommends bootstrapping instead. In the next section, we propose a wild bootstrap algorithm (Wu, 1986) for the null distribution of $T_{n}$ following Hardle and Mammen (1993). Note that one may also consider an empirical version of the proposed test statistic evaluated at the paired time points (see Web Appendix A for an example); we focus on (6) throughout this article.

### 3.4 Approximate null distribution of $T_{n}$ via a wild bootstrap

Denote the *l*‐th bootstrap sample as $\left\{Y_{\mathrm{ij}}^{\left(l\right)}:i=1,\ldots ,n,j=1,\ldots ,m_{i},t_{\mathrm{ij}}\in T\right\}$, where $Y_{\mathrm{ij}}^{\left(l\right)}=\hat{\mu }(t_{\mathrm{ij}})+X_{i}^{\left(l\right)}(t_{\mathrm{ij}})+\varepsilon_{\mathrm{ij}}^{\left(l\right)}$ for the original time points $t_{\mathrm{ij}}$. Let $\hat{\mu }(t)$ be the estimated smooth mean function, $X_{i}^{\left(l\right)}(t_{\mathrm{ij}})$ be subject trajectories generated from the estimated null model in (4), and $\varepsilon_{\mathrm{ij}}^{\left(l\right)}$ be simulated residuals using the estimated measurement error in Section 3.2. The test statistic, $T_{n}^{\left(l\right)}$, can be calculated from the resulting bootstrap sample, and the process is repeated to obtain an approximation of the null distribution of $T_{n}$. If the observed statistic is large compared to the null approximation, then reject $H_{0}$. This “wild” bootstrap procedure (Wu, 1986) is outlined in Algorithm 1 and is valid in the regression function setting (Hardle and Mammen, 1993).

**Table:** 

Algorithm 1 Parametric bootstrap for null distribution of $T_{n}$
1: **for** l∈{1,…,L} **do**
2: Generate $X_{i}^{\left(l\right)}(t_{\mathrm{ij}})=b_{0i}^{\left(l\right)}+b_{1i}^{\left(l\right)}t_{\mathrm{ij}}$ from $\left(b_{i0}^{\left(l\right)},b_{1i}^{\left(l\right)}{\;)}^{T}∼N(0,{\hat{V}}_{0}\right)$ for i∈{1,…,n}, where ${\hat{V}}_{0}$ is the estimated parameter matrix under the null hypothesis in (4).
3: Sample $\varepsilon_{\mathrm{ij}}^{\left(l\right)}∼N(0,{\hat{\sigma }}^{2})$ for i∈{1,…,n} and $j\in \{1,\ldots ,m_{i}\}$, where ${\hat{\sigma }}^{2}$ is the measurement error estimated under the alternative model in Section 3.2.
4: Define the *l*‐th bootstrap dataset as $Y_{\mathrm{ij}}^{\left(l\right)}=\hat{\mu }(t_{\mathrm{ij}})+X_{i}^{\left(l\right)}(t_{\mathrm{ij}})+\varepsilon_{\mathrm{ij}}^{\left(l\right)}$.
5: Estimate and subtract the mean function for the bootstrap data, $\mu^{\left(l\right)}(t)$.
6: Fit the *l*‐th bootstrap dataset with model (4) and estimate ${\hat{G}}_{0}^{\left(l\right)}$.
7: Fit the *l*‐th bootstrap dataset with model (5) and calculate ${\hat{G}}_{A}^{\left(l\right)}$.
8: Calculate the test statistic $T_{n}^{\left(l\right)}=||{\hat{G}}_{A}^{\left(l\right)}-K{\hat{G}}_{0}^{\left(l\right)}|{|}_{\mathrm{HS}}$.
9: **end for**
10: Calculate *p*‐value$=L^{-1}\sum_{l=1}^{L}𝕀(T_{n}^{\left(l\right)}>T_{n})$, where 𝕀 is an indicator function with value 1 if the condition is true, and 0 otherwise.

## 4 Implementation

First, estimate the smooth mean μ(t) using thin plate regression splines (Wood, 2003) using the gam function in the R package mgcv (Wood, 2017), and subtract from the data. The null model in (4) is a standard random effects model that can be estimated using the lme function in the R package nlme (Pinheiro et al., 2017). For the least squares expression in (5) to smooth the alternative and null covariance estimates, we use H=10 cubic B‐splines per axis with equally‐spaced interior knots. The choice of 10 B‐splines balances performance and computational speed, see Web Appendix B for a sensitivity study. While the number of splines needs only be sufficiently large, additional splines may be needed if the data is known or observed to be highly wiggly. Cross‐validation or Aikaike information criterion (AIC) may be used for a formal selection (see Wood (2003) for discussion).

## 5 Extensions

### 5.1 Smooth covariance

Any smooth parametric covariance function can be tested using the proposed procedure, with modification to the null model and bootstrap algorithm. For example, consider the stationary Gaussian or quadratic exponential covariance function $G_{0}(t,t^{\prime })=\theta e^{-h^{2}/\delta^{2}}$, where $h=|t-t^{\prime }|$, and (θ,δ) are parameters to be estimated. The null model can be estimated using likelihood‐based methods, and bootstrap data generated as $Y_{i}^{\left(l\right)}={\hat{\mu }}_{i}+{\hat{V}}_{0i}^{(l)\frac{1}{2}}z+\varepsilon_{i}^{\left(l\right)}$, where ${\hat{\mu }}_{i}$ is the estimated mean vector of length $m_{i}$, ${\hat{V}}_{0i}^{\left(l\right)}$ is the estimated null covariance matrix defined by $\left(\hat{\theta },\hat{\delta }\right)$, $X^{\frac{1}{2}}$ is the square root matrix where $X^{\frac{1}{2}}X^{\frac{1}{2}}=X$, z is an $m_{i}$‐length vector of independent samples from a standard normal distribution, and $\varepsilon_{i}^{\left(l\right)}$ is an independent vector of residuals from $N(0,{\hat{\sigma }}^{2})$.

## 6 Simulation Study

We conduct a simulation study to evaluate performance of the proposed *bootstrap* test and two competing methods, described in Section 6.1, for testing the hypothesis in (3) that the covariance has a quadratic polynomial form. Data are generated as

(7) $$\begin{array}{cc} Y_{\mathrm{ij}} & =\mu (t_{\mathrm{ij}})+X_{i}(t_{\mathrm{ij}})+\varepsilon_{\mathrm{ij}}\\ X_{i}(t_{\mathrm{ij}}) & =b_{0i}+b_{1i}t_{\mathrm{ij}}+\Delta z_{i}(t_{\mathrm{ij}}), \end{array}$$

for i=1,…,n subjects and $j=1,\ldots ,m_{i}$ observations per subject. The scalar, Δ, controls the magnitude of deviation from the null model. The mean, μ(t), is set to 0 and the residuals are distributed $\varepsilon_{\mathrm{ij}}∼N(0,1)$, independent of $X_{i}$. Random intercepts and slopes are sampled from a bivariate normal distribution with zero mean, $\mathrm{Var}(b_{i0})=\mathrm{Var}(b_{i1})=1$ and $\mathrm{Cov}(b_{i0},b_{i1})=-0.5$, independent of the non‐linear function $z_{i}$, defined below. The $t_{\mathrm{ij}}$ are observed on a grid of 80 equally spaced points in [−1,1]. If $m_{i}=80$, the subject is observed at all points and if $m_{i}<80$, observed time points are uniformly sampled for each subject from the 80 possible points. Tuning parameters are selected as described in Section 4. Consider a factorial combination of the following factors:

1. **Observations per subject** ($m_{i}=m$): (a) m=80, (b) m=40, (c) m=20, (d) m=10
2. **Deviation from the null model**:
  - **Quadratic:** $z_{i}(t)=b_{2i}t^{2},b_{2i}∼N(0,1)$
  - **Trigonometric:** $z_{i}(t)=\sum_{k=1}^{2}\xi_{\mathrm{ik}}\psi_{k}(t),\{\psi_{1}(t),\psi_{2}(t)\}=\{\sin(2\pi t),\text{sin}(4\pi t)\},\xi_{\mathrm{ik}}∼N(0,\lambda_{k})$, $\lambda_{1}=\lambda_{2}=1$.

For each factor combination, we use L=1000 bootstrap samples per dataset and consider n=100 and 500 subjects, and n=50 for the m=80 setting only. Performance is evaluated in terms of the empirical type I error rate (size) for nominal levels α=0.05 and 0.10 based on 5000 simulated datasets, and power at the α=0.05 level with 1000 simulated datasets. Results are presented in terms of deviation from the null, defined as $\Delta^{2}\int \mathrm{Var}\{z_{i}(t)\}/\mathrm{Var}\{X_{i}(t)\}\mathrm{dt}$.

### 6.1 Competing methods

As discussed in Section 1, we are unaware of any existing methods for testing covariance that can be applied to all functional or longitudinal data settings. In this subsection, we describe two testing methods that can be applied to specific scenarios of the hypothesis test in (3).

#### 6.1.1 Direct test

Consider the case where covariance under the alternative hypothesis has a known, parametric form so the null model for $X_{i}$ is nested within the alternative model. In essence, test if a more complex covariance better explains the data than the null covariance. For the quadratic polynomial covariance, an alternative may be $G_{A}(t,t^{\prime })=\sigma_{0}^{2}+\sigma_{01}(t+t^{\prime })+\sigma_{1}^{2}{\mathrm{tt}}^{\prime }+\sigma_{2}^{2}t^{2}t^{\prime 2}$. Then the alternative model can be written as

(8) $$\begin{array}{cc} X_{i}(t_{\mathrm{ij}}) & =b_{0i}+b_{1i}t_{\mathrm{ij}}+b_{2i}t_{\mathrm{ij}}^{2}\\ b_{i} & =(b_{0i},b_{1i},b_{2i}{\;)}^{T}∼N\left(0,\left[\begin{array}{ccc} \sigma_{0}^{2} & \sigma_{01} & 0\\ \sigma_{01} & \sigma_{1}^{2} & 0\\ 0 & 0 & \sigma_{2}^{2} \end{array}\right]\right). \end{array}$$

Note that this is the model for the quadratic deviation setting in the simulation study. Like the null model, (8) can be estimated using the lme function in the R package nlme (Pinheiro, et al., 2017). The hypothesis test is equivalent to testing if $b_{2i}=0$, or $H_{0}:G(t,t^{\prime })=G_{0}(t,t^{\prime })\Leftrightarrow \sigma_{2}^{2}=0$ versus $H_{A}:G(t,t^{\prime })\neq G_{0}(t,t^{\prime })\Leftrightarrow \sigma_{2}^{2}>0$.

Testing zero‐value variance components is a non‐standard problem because the null hypothesis is on the boundary of the parameter space. Self and Liang (1987) derive the asymptotic null distribution of the likelihood ratio test (LRT) for this setting as a mixture of chi‐squared distributions. Crainiceanu and Ruppert (2004) derive the exact finite sample null distribution for the (R)LRT of mixed models with one random effect, and Greven et al. (2008) extend this approach to models with multiple random effects using pseudolikelihood. Because of the limited sample size in our simulation study, we use the finite sample null distribution from Greven et al. (2008), which can be preformed efficiently using the exactRLRT function in the R package RLRsim (Schiepl and Bolker, 2016).

#### 6.1.2 Multivariate test

The Zhong et al. (2017) test for high‐dimensional multivariate data can be applied to functional data with a common design. Consider a repeated measures model $Y_{i}=\mu +\varepsilon_{i}$, where $Y_{i}=(Y_{i1},\ldots ,Y_{\mathrm{im}}{\;)}^{T}$ is a vector of responses, μ is a mean vector of length *m*, and residuals are distributed $\varepsilon_{i}∼N(0,G)$. Denote $\theta_{0}$ as the parameter vector defining the covariance matrix under the null hypothesis, $G_{0}$. Let $G_{A}$ be the alternative unstructured covariance.

Based on the squared‐Frobenius distance between the null and alternative covariances, $\delta (\theta_{0})=\mathrm{tr}(G_{A}-G_{0}{\;)}^{2}$, Zhong et al. (2017) propose the test statistic $\Lambda_{n}={\hat{T}}_{n}-{\hat{J}}_{n3}$, where ${\hat{T}}_{n}$ is an unbiased estimator for $\delta (\theta_{0})$ and ${\hat{J}}_{n}$ adjusts for errors in the estimation of $\theta_{0}$. The hypothesis test in (3) can be conducted by testing if $\Lambda_{n}$ is significantly larger than 0. With some assumptions on the covariance structure, the asymptotic normal and fixed sample weighted‐chi square null distributions can be determined for any parametric covariance, and we provide derivations for the quadratic polynomial covariance in Web Appendix C. In our simulation study, the *multivariate* test can only be applied to the dense m=80 case, and we use 10,000 samples to approximate the fixed sample null distribution. In Web Appendix D, we also consider performance of the *multivariate* test in less‐ideal settings with small *m* and unequally‐spaced data.

#### 6.1.3 Limitations of the competing methods

While both competitors utilize test statistics with known null distributions, these tests only apply to limited scenarios. The *direct* test applies when the alternative is known, parametric, and a superset of the null model. The *multivariate* test only applies to data with a common design and assumes an unstructured covariance that does not account for smoothness. Thus, the *bootstrap* test is expected to be more powerful than the *multivariate* test for testing functional data.

### 6.2 Simulation results

Table 1 reports the empirical type I error rates for all three methods. As the *multivariate* test requires a common sampling design, it can only be applied to the m=80 setting. We report only the fixed‐sample weighted chi‐squared distribution; results for the asymptotic normal distribution were similar and are presented in Web Appendix D. All three methods have empirical levels close to the nominal levels, although both the *bootstrap* and *direct* tests can be slightly conservative for several settings.

**Table 1:** Estimated type I error rates for the *bootstrap*, *direct*, and *multivariate* tests at the nominal α=0.05 and 0.10 levels based on 5000 datasets, by number of subjects (*n*) and observations per subject (*m*). The standard error was 0.003 and 0.004 for α=0.05 and α=0.10, respectively. The *multivariate* test is applicable for only the dense m=80 setting

	**Bootstrap**		**Direct**		**Multivariate**		
n	m	α=0.05	α=0.10	α=0.05	α=0.10	α=0.05	α=0.10
100	10	0.059	0.126	0.044	0.086	n/a	n/a
	20	0.045	0.105	0.049	0.098	n/a	n/a
	40	0.042	0.093	0.049	0.102	n/a	n/a
	80	0.042	0.091	0.045	0.096	0.053	0.103
500	10	0.047	0.105	0.049	0.096	n/a	n/a
	20	0.050	0.103	0.049	0.096	n/a	n/a
	40	0.050	0.100	0.043	0.090	n/a	n/a
	80	0.044	0.093	0.046	0.096	0.053	0.105

**Figure 1:**
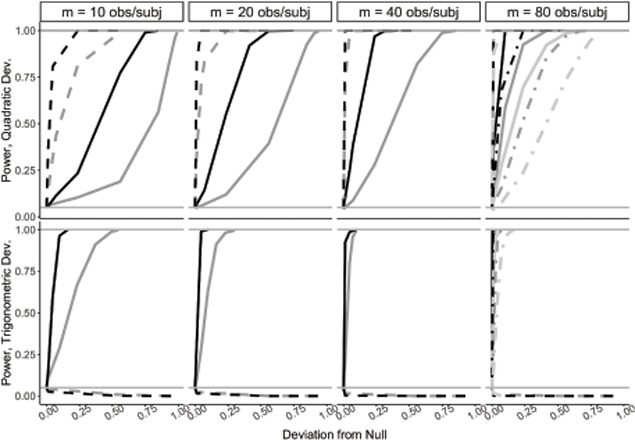
Power under the quadratic (top) and trigonometric (bottom) deviations from the null, by number of observations per subject, *m*. Shown are: *bootstrap* test (solid), *multivariate* test (long and short‐dash), and *direct* test (long‐dash), for n=50 (light gray), n=100 (dark gray), and n=500 (black) subjects. The *multivariate* test is not applicable when m<80.

Figure 1 presents simulation results for the quadratic and trigonometric deviations from the null, by number of observations per subject, *m*. For all methods, power increases with sample size, particularly as data are more densely sampled and the covariance is better estimated. As expected, power depends on how closely the true model matches the specific alternative assumed by the test. The *bootstrap* test outperforms the *multivariate* test for all settings because of the more specific form of its alternative, and has higher power than the *direct* test when the *direct* test has misspecified the alternative (trigonometric deviation). Conversely, the *direct* test has higher power when the parametric alternative is correctly specified (quadratic deviation). Both the *bootstrap* and *multivariate* tests are better able to detect the trigonometric deviation because the covariance more obviously deviates from the null model. Overall, the *bootstrap* test performs well in most settings, except when the dataset is small and deviation from the null is small. For example, when n=100 and m=10, the test is underpowered for the quadratic deviation when signal size is small.

In terms of computational speed, the *bootstrap* test is, unsurprisingly, significantly slower than the competitor methods. A personal laptop with a 2.9 GHz processor took 1–7 min to run a single iteration, compared to 1 and 0.2 s for the *direct* and *multivariate* tests, respectively, for the null model with 100 subjects. Reducing the density of inputted data or *L* number of bootstrap samples can decrease computational time, with some loss of power.

## 7 Applications

### 7.1 Diffusion tensor imaging

**Figure 2:**
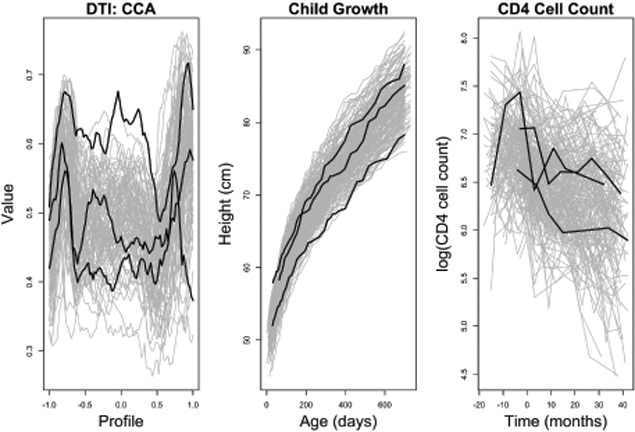
(left) Diffusion tensor imaging (DTI) of corpus callosum (CCA) baseline tract profiles from 99 multiple sclerosis patients. (middle) Height measurements (cm) for 215 children from 0 to 729 days after birth. (right) Log‐transformed CD4 cell counts from 208 subjects for −18 to 52 months since seroconversion. On each plot, three example trajectories are highlighted in black.

We first consider a dataset of diffusion tensor imaging (DTI) of intracranial white matter microstructure with dense, common sampling design for a group of normal and multiple sclerosis patients. Images of the white matter are depicted with tract profiles shown in Figure 2 and available in the R package refund (Goldsmith et al., 2016); see Reich et al. (2010) for study details. Goldsmith et al. (2011) consider this dataset for modeling multiple sclerosis disease status, concluding that inclusion of the tract profile as a functional predictor improves model performance compared to a subject‐specific average of the profile. Note that a subject‐specific average is equivalent to the subject‐specific intercept in the null model in (4). We evaluate this conclusion formally by testing if a quadratic polynomial covariance is sufficient for modeling the tract profiles, using the *bootstrap*, *multivariate*, and *direct* tests.

We focus on the baseline tract profiles of the corpus callosum (CCA), associated with cognitive function, for multiple sclerosis patients, observed on a dense, regular grid of 93 points. After removing subjects with missing observations, the dataset has profiles from 99 subjects, for a total of 9207 observations. Tuning parameters are selected as described in Section 4. The observed test statistic for the *bootstrap* test is $T_{n}=0.071$ corresponding to p<0.001. The *direct* test yields an RLRT statistic of 1160.6 corresponding to $p<1\times 10^{-16}$. The *multivariate* test yields an observed test statistic of $\Lambda_{n}=0.058$, corresponding to p<0.001 for both the weighted chi‐squared and asymptotic normal distributions. All three tests support the conclusion that a quadratic polynomial covariance is inadequate for the data, and that a functional method should be used.

### 7.2 Child growth measurements

Next, consider the CONTENTS child growth dataset from Lima, Peru (Xiao et al., 2018). The dataset contains irregularly sampled height measurements for 215 children covering 0 to 729 days after birth, for a total of 8839 observations (20–50 observations per subject, observed at different time points), shown in Figure 2. Subject trajectories predicted using functional principal components analysis, shown in Xiao et al. (2018), exhibit curvature not captured by a linear parametric model, suggesting that a functional approach is necessary for the data. We consider this observation formally by testing the quadratic polynomial covariance for the growth data using the *bootstrap* and *direct* tests.

The observed test statistic for the *bootstrap* test is $T_{n}=494.13$, corresponding to p=0.031, while the RLRT statistic from the *direct* test is 2205.8, corresponding to p<0.001. Both tests indicate that the parametric quadratic polynomial covariance is not sufficient for the data, and a functional approach should be used instead.

### 7.3 CD4 count data

Last, we consider the motivating example of CD4 cell counts described in Section 1 by conducting a formal test of the quadratic polynomial covariance using the *bootstrap* and *direct* tests. The dataset is available in the R package refund (Goldsmith et al., 2016) and includes cell counts from −18 to 52 months since seroconversion; we log‐transform the counts to stabilize variability. We consider only subjects with at least 5 observations and who have log‐transformed cell counts greater than 4, for a total of 1402 observations from 208 subjects (5–11 observations per subject). The cleaned and log‐transformed data are shown in Figure 2.

Because data are sparser than the settings considered in the full study, we conduct a small simulation study to check the size and power of the tests. Simulated data are generated as $Y_{\mathrm{ij}}=X_{i}(t_{\mathrm{ij}})+\varepsilon_{\mathrm{ij}}$, where $X_{i}(t_{\mathrm{ij}})$ is defined below, $t_{\mathrm{ij}}$ are the time points in the original dataset, and $\varepsilon_{\mathrm{ij}}∼N(0,{\hat{\sigma }}^{2})$, where ${\hat{\sigma }}^{2}$ is the estimated error variance under the alternative model. The random function $X_{i}(t)$ is generated from a multivariate normal distribution with zero‐mean and covariance $G=(1-\delta ){\hat{G}}_{0}+\delta {\hat{G}}_{A}+rG_{1},$ where ${\hat{G}}_{0}$ and ${\hat{G}}_{A}$ are the estimated covariance matrices from the null and alternative model, respectively, δ∈[0,1] controls the contribution of the null and alternative covariances, and $G_{1}$ is the matrix generated from the first three eigenfunctions and eigenvalues of (${\hat{G}}_{A}-{\hat{G}}_{0}$), with magnitude controlled by r≥0. Note that when δ=r=0, G is the null covariance, and when δ=1 and r=0, G is the alternative covariance. To show how power changes with deviation from the null model, let δ=1 when r>0. Since the *bootstrap* test is likely to be underpowered due to sparsity of the data, we also simulate data with double the number of subjects or double the observations per subject. Additional subjects were generated using the same set of observed time points, while additional observations were added by uniformly sampling from the non‐observed time points for each subject.

**Table 2:** Estimated type I error rates for the *bootstrap* and *direct* tests at the nominal α=0.05 and 0.10 levels based on 5000 datasets, for data based on the standard CD4 dataset, dataset with double the number of subjects, and dataset with double the observations per subject. The standard error was 0.003 and 0.004 for α=0.05 and α=0.10, respectively

	**Bootstrap**		**Direct**	
	α=0.05	α=0.10	α=0.05	α=0.10		
Standard dataset	0.057	0.113	0.046	0.010		
Double subjects	0.056	0.108	0.047	0.095		
Double observations	0.046	0.101	0.050	0.100		

**Figure 3:**
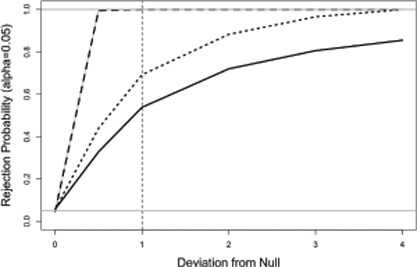
Power for the *bootstrap* (black) and *direct* (gray) tests for data based on the standard CD4 dataset (solid), dataset with double the number of subjects (short dash), and dataset with double the observations per subject (long dash). The vertical dashed line indicates the effective power of the tests, where data is simulated directly from the estimated alternative covariance (δ=1,r=0). From left to right, the settings for (δ,r) are (0,0),(0.5,0),(1,0),(1,1),(1,2), and (1,3).

Table 2 gives the empirical type I error rates based on 5000 simulated datasets, and Figure 3 shows the power from 1000 datasets, for the *bootstrap* and *direct* tests. The *bootstrap* test is underpowered for the true CD4 dataset due to small sample size, and doubling the number observations per subject resolves this problem.

The observed test statistic for the *bootstrap* test is $T_{n}=5.025$ corresponding to p=0.100, while the *direct* test yields an RLRT statistic of 2.704 corresponding to p=0.0428. While only the *direct* test indicates that the quadratic polynomial covariance is not sufficient for the data, Figure 3 shows that the *bootstrap* test is underpowered, suggesting that a more complex covariance may still be necessary for the data.

## 8 Concluding Remarks

In this article, we propose a smoothing‐based goodness‐of‐fit test of covariance for functional data. We focus on the specific case of testing a quadratic polynomial covariance induced by a linear random intercept and slope model, as motivated by a dataset of CD4 cell counts used to monitor HIV+ patients. Our proposed method can be used to formally test a linear random (or mixed) effects model against a typical functional data approach, and fills a gap in the testing of longitudinal and functional data methods. The proposed *bootstrap* test can be applied to functional data with either dense common or irregular sampling design, and performs well in simulation studies. Limitations of the method are (a) slow computational speed, and (b) low power for very small datasets with small deviation from the null.

## Supporting information

Web Appendices referenced in Sections 3.3, 4, 6.1.2, 6.2, and 6.3 are available with this article at the Biometrics website on the Wiley Online Library. R code implementing the *bootstrap*, *direct*, and *multivariate* tests for the quadratic polynomial covariance is available with this article at the Biometrics website on the Wiley Online Library.

Supplementary Data S1.

Supplementary R Code S1.
