# Supplementary material for: A smoothing‐based goodness‐of‐fit test of covariance for functional data
Source: Biometrics. 2019 Apr 6;75(2):562–71. doi: 10.1111/biom.13005 (PMC6526086; doi:10.1111/biom.13005)
Supplement: Supplementary file 1 — Supplementary Data S1. [file BIOM-75-562-s001.pdf]

Biometrics 000, 1–4 DOI: 000
000 0000

Web-based Supplementary Materials for "A Smoothing-based Goodness-of-Fit Test of Covariance for Functional Data"

Stephanie T. Chen*, Luo Xiao, and Ana-Maria Staicu

Department of Statistics, North Carolina State University, Raleigh, North Carolina, U.S.A.

**email:* stchen3@ncsu.edu

Web Appendix A: Weighting for Non-Uniform Sampling

In this section we describe an empirical extension of the test statistic proposed in Section 3.3 of the main paper that weights by the frequency of observed time points

$$T_n' = \int\int \{\mathcal{G}_A(s,t) - \mathcal{K}\mathcal{G}_0(s,t)\}^2 dQ_n(s,t), \qquad (1)$$

where i indexes subjects, j indexes observations per subject, $\mathcal{G}_A$ is the alternative covariance, $\mathcal{K}\mathcal{G}_0$ is the smoothed null covariance, as described in the main paper, $Q_n(s,t) = \frac{1}{N}\sum_{i=1}^n \sum_{j\neq j'} \mathbf{1}_{T_{ij}<s, T_{ij'}<t}$ is the weighting function, and $N = \sum_{i=1}^n m_i(m_i-1)$, .

This extended statistic accounts for non-uniformity of sampling points, but is more tedious to calculate. To compare performance of the standard statistic, T_n, with T_n', we conduct a small simulation study. Following Section 6, we will consider both quadratic and trigonometric deviations for $n = 100$ subjects and $m = 10$ observations per subject. This setting is most likely to be impacted by non-uniform sampling. Points t_{ij} are selected uniformly as in the main study, and non-uniformly by weighting points by their normal distribution density so points at the center of the interval are more likely to be observed.

Web Table 1 gives the type I error rates for both statistics, which have similar and slightly inflated error rates due to the small sample size. Web Figure 1 shows the power of the two statistics with uniform and non-uniform sampling. The extended statistic T_n' is more powerful than the standard statistic for all settings. For the quadratic deviation, T_n' has <5% higher power than T_n regardless of the sampling process. For the trigonometric deviation, T_n' improves power by 1-5% with uniform data but can improve power by 10-20% with non-uniform data. Both statistics are valid regardless of sampling scheme, but the extended T_n' statistic has better overall performance by accounting for the empirical frequency of time points.

Web Appendix B: Sensitivity

In this section we provide a small simulation study on the sensitivity of the *bootstrap* test to the number of basis functions used for the alternative model fit in Equation (5) of the main paper. We repeat the simulation study in Section 6 for $n = 100$ subjects using 7 bivariate tensor-product regression splines with equally-spaced interior knots (default = 10 splines), based on 5000 simulated datasets for type I error rate (Web Table 2) and 1000 for power (Web Figure 2). Overall, the *bootstrap* test does not appear to be sensitive to the number of basis functions. For 7 basis functions, the test is slightly more powerful for the quadratic deviation and significantly less powerful for the trigonometric deviation. The test ran approximately 15-40% faster with 7 basis functions compared to the default 10 and 15-40% slower with 13 basis functions (not shown) depending on the number of observations per subject.

Web Appendix C: Multivariate Test for Quadratic Polynomial Covariance

In this section we outline derivations for the form of the Zhong et al. (2017) *multivariate* test for the quadratic polynomial covariance. As stated in the main paper, consider a repeated measures model $\boldsymbol{Y}_i = \boldsymbol{\mu} + \boldsymbol{\epsilon_i}$, where $\boldsymbol{Y}_i = (Y_{i1}, \ldots, Y_{im})^T$ is a vector of responses, $\boldsymbol{\mu}$ is a mean vector of length m, and residuals are distributed $\boldsymbol{\epsilon}_i \sim N(\mathbf{0}, \boldsymbol{G})$. Denote $\boldsymbol{\theta}_0$ as the q-dimensional parameter vector defining the covariance matrix under the null hypothesis, $\boldsymbol{G}_0$. Let $\boldsymbol{G}_A$ be the unstructured covariance under the alternative hypothesis, assumed to be symmetric. The test statistic Λ_n is defined as

$$\begin{aligned}
\Lambda_n &= \hat{T}_n - \hat{J}_{n3} \\
\hat{T}_n &= \frac{1}{C_n^2}\sum_{i<i'}^{n}(\hat{\boldsymbol{\epsilon}}_i^T\hat{\boldsymbol{\epsilon}}_{i'})^2 - \frac{2}{n}\sum_{i=1}^{n}\hat{\boldsymbol{\epsilon}}_i^T\hat{\boldsymbol{G}}_0\hat{\boldsymbol{\epsilon}}_i + tr(\hat{\boldsymbol{G}}_0^2) \\
\hat{J}_{n3} &= \frac{1}{n}\Big(\frac{1}{C_n^2}\sum_{i<i'}^{n}\hat{\boldsymbol{e}}_i^T\hat{\boldsymbol{\Omega}}_0\hat{\boldsymbol{e}}_{i'} - \frac{1}{n}\sum_{i=1}^{n}\hat{\boldsymbol{e}}_i^T\hat{\boldsymbol{\Omega}}_0\hat{\boldsymbol{e}}_i\Big),
\end{aligned}$$

where $\hat{T}_n$ is an unbiased estimator for $\delta(\boldsymbol{\theta}_0)$ and $\hat{J}_n$ is an adjustment for error in estimating $\boldsymbol{\theta}_0$. The covariance matrix can be solved for consistently as $\hat{\boldsymbol{\theta}}_0 = \arg\min_{\boldsymbol{\theta}} tr\{(\hat{\boldsymbol{G}}_A - \boldsymbol{G}_0)^2\}$, where $\hat{\boldsymbol{G}}_A = n^{-1}\sum_{i=1}^{n} \hat{\boldsymbol{\epsilon}}_i^T \hat{\boldsymbol{\epsilon}}_i$, $\hat{\boldsymbol{\epsilon}}_i = \boldsymbol{Y}_i$, and $C_n^k = \frac{n!}{(n-k)!k!}$. For $u = 1, 2, 3$, define $\hat{\boldsymbol{e}}_i = (\hat{e}_{i,1}, \ldots, \hat{e}_{i,q}^T)$, $\hat{e}_{i,k} = \hat{\boldsymbol{\epsilon}}_i^T \boldsymbol{B}_{k,0} \hat{\boldsymbol{\epsilon}}_i$, $\boldsymbol{B}_{k,u} = \frac{\partial \boldsymbol{G}_0}{\partial \theta_{0k}} \boldsymbol{G}_0^u$, θ_{0k} is the k-th entry of $\boldsymbol{\theta}_0$, $\boldsymbol{\Omega}_0 = \boldsymbol{V}_0^{-1}$, and $\boldsymbol{V}_{\boldsymbol{u}} = \{tr(\boldsymbol{B}_{c,u}\boldsymbol{B}_{d,u})\}_{[c,d]}$.

With some assumptions on the covariance structure, the asymptotic null distribution of Λ_n is $\sigma_{\Lambda_n,0}^{-1}\Lambda_n \xrightarrow{d} N(0,1)$, where $\sigma_{\Lambda_n,0}^2 = 2\xi_1^2/C_n^2$ and $\xi_1^2 = tr^2(\boldsymbol{G}_0^2) - tr(\boldsymbol{G}_0) + 2tr\{(\boldsymbol{\Omega}_0\boldsymbol{V}_1)^2\} - 4tr(\boldsymbol{\Omega}_0\boldsymbol{V}_2)$. The fixed-sample null distribution is a weighed chi-squared distribution $n\Lambda_n \xrightarrow{d} \sum_{k=1}^{\infty} \lambda_k(\chi_{1,k}^2 - 1)$, where λ_k are the eigenvalues from the kernel $h(\boldsymbol{\epsilon}_i, \boldsymbol{\epsilon}_j) = (\boldsymbol{\epsilon}_i^T\boldsymbol{\epsilon}_j)^2 - \boldsymbol{\epsilon}_i^T\boldsymbol{\Sigma}\boldsymbol{\epsilon}_j + tr(\boldsymbol{G}_A^2) - \boldsymbol{Q}^T(\epsilon_i)\boldsymbol{\Omega}_0\boldsymbol{Q}(\boldsymbol{\epsilon}_j)$, where $\boldsymbol{Q}(\boldsymbol{\epsilon}_i) = [Q_1(\boldsymbol{\epsilon}_i), \ldots, Q_q(\boldsymbol{\epsilon}_i)]$ and $Q_k(\boldsymbol{\epsilon}_i) = e_{i,k} - tr(\boldsymbol{B}_{k,0}\boldsymbol{G}_A)$. As sampling from such a distribution is difficult, a Satterthwaite's approximation can be used instead, defined as $g_1\chi_{g_2}^2 - g_3$, where $g_1 = \sum_k \lambda_k^2/\sum_k \lambda_k$, $g_2 = (\sum_k \lambda_k)^2/\sum_k \lambda_k^2$, $g_3 = \sum_k \lambda_k$, $\sum_k \lambda_k = tr^2(\boldsymbol{G}_0) + tr(\boldsymbol{G}_0^2) - 2tr(\boldsymbol{\Omega}_0\boldsymbol{V}_1)$, and $\sum_k \lambda_k^2 = 2\xi_1^2$.

Next, consider the quadratic polynomial covariance in Equation (2) of the main paper. The null covariance matrix is $\boldsymbol{G}_0 = \sigma_0^2\boldsymbol{1}\boldsymbol{1}^T + \sigma_{01}(\boldsymbol{1}\boldsymbol{t}^T + \boldsymbol{t}\boldsymbol{1}^T) + \sigma_1^2\boldsymbol{t}\boldsymbol{t}^T + \sigma^2\boldsymbol{I}_m$ for parameters $\boldsymbol{\theta}_0 = (\sigma_0^2, \sigma_{01}, \sigma_1^2, \sigma^2)^T$, and $\boldsymbol{1}$ is a m-length vector of ones. Because the mean is assumed to be zero, $\hat{\boldsymbol{\epsilon}}_i = \boldsymbol{Y}_i$, $\boldsymbol{B}_{1,u} = \boldsymbol{1}\boldsymbol{1}^T\boldsymbol{G}_0^u$, $\boldsymbol{B}_{2,u} = (\boldsymbol{1}\boldsymbol{t}^T + \boldsymbol{t}\boldsymbol{1}^T)\boldsymbol{G}_0^u$, $\boldsymbol{B}_{3,u} = \boldsymbol{t}\boldsymbol{t}^T\boldsymbol{G}_0^u$, $\boldsymbol{B}_{4,u} = \boldsymbol{G}_0^u$, $\boldsymbol{V}_{\boldsymbol{u}} = [tr(\boldsymbol{B}_{c,u}\boldsymbol{B}_{d,u})]_{[c,d]}$, for $c, d = 1, 2, 3, 4$. Verifying the assumptions for this covariance is non-trivial because of the four parameters involved, however, simulations with large m result in a null distribution for Λ_n that closely matches the derived asymptotic normal distribution.

Web Appendix D: Multivariate Test for few and unequally-spaced data

In this section we consider performance of the *multivariate* test when the data are observed at fewer and unequally-spaced points. The test was intended for dense, high-frequency data and our goal here is to understand its performance in less ideal settings. We conduct a simulation study with the same factorial combinations as in Section 6 of the main paper. However, let $t \in [-1, 1]$ be observed at m equally or unequally-spaced points. For the unequally-spaced settings, randomly sample the common points from a continuous Uniform$(-1, 1)$ distribution. Empirical type I error rates are reported for 5000 simulated datasets in Web Table 3, and rejection probabilities for 1000 datasets in Web Figure 3. In terms of type I error rate, the fixed sample chi-squared distribution maintains the nominal levels for all settings, while the asymptotic normal distribution is elevated, particularly when the observations are unequally-spaced. Power is higher for equally-spaced data with the quadratic deviation, particularly when the data are sparse, but similar for the equally- and unequally-spaced data with the trigonometric deviation.

Web Table 1. Estimated type I error rates for the standard (T_n) and extended (T'_n) *bootstrap* test statistics with uniform or non-uniform distributed sampling points. The nominal $\alpha = 0.05$ and 0.10 levels are based on 5000 simulated datasets for $n = 100$ subjects and $m = 10$ observations per subject.

	Uniform		Non-Uniform	
	$\alpha = 0.05$	$\alpha = 0.10$	$\alpha = 0.05$	$\alpha = 0.10$
T_n	0.059	0.126	0.054	0.126
T'_n	0.059	0.117	0.054	0.120

Web Figure 1. Power for the *bootstrap* test under the quadratic (top) and trigonometric (bottom) deviations from the null for $n = 100$ subjects and $m = 10$ observations per subject. Shown are the standard T_n statistic (black, solid) and extended T'_n statistic (gray, dashed) for the quadratic deviation (top) and trigonometric deviation (bottom). Note that the x-axis range differs between the quadratic and trigonometric deviations.

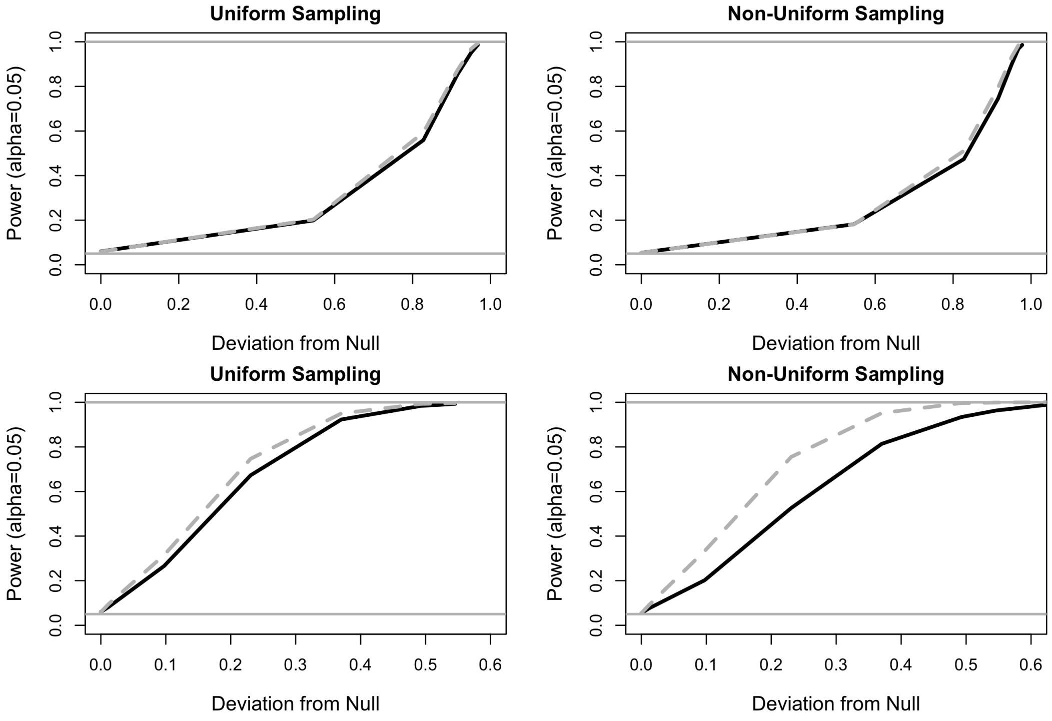

Web Table 2. Estimated type I error rates for the *bootstrap* test at the nominal $\alpha = 0.05$ and 0.10 levels using 10 (default) or 7 basis functions for the alternative model fit in (6) based on 5000 simulated datasets for $n = 100$ subjects, by number of observations per subject (m).

# basis	m	$\alpha = 0.05$	$\alpha = 0.10$
10	10	0.059	0.126
7		0.048	0.102
10	20	0.45	0.105
7		0.044	0.096
10	40	0.042	0.093
7		0.044	0.097
10	80	0.042	0.091
7		0.054	0.105

Web Figure 2. Power for the *bootstrap* test under the quadratic (top) and trigonometric (bottom) deviations from the null, by number of observations per subject (m). Shown are: (default) 10 basis functions (black, solid) and 7 basis function (gray, dashed). Note that the x-axis range differs between the quadratic and trigonometric deviations.

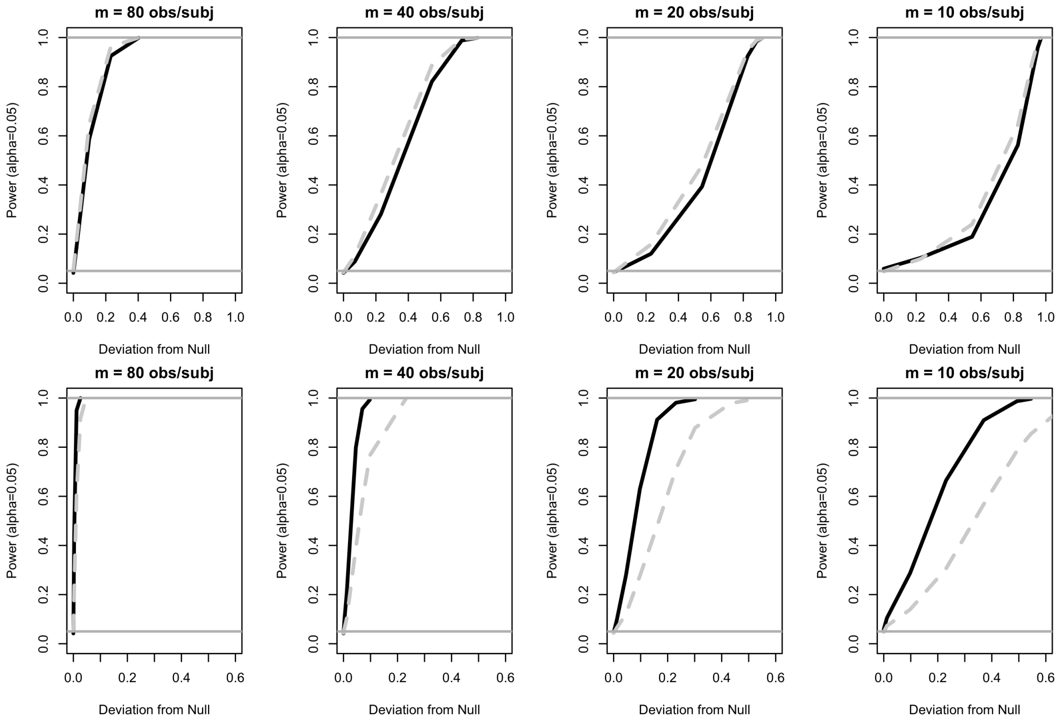

Web Table 3. Empirical type I error rates for the *multivariate* test at the nominal $\alpha = 0.05$ level based on 5000 datasets, for the asymptotic normal (Z) and weighted chi-squared ($\omega\chi^2$) distributions, and equally- and unequally-spaced data.

		equally-spaced		unequally-spaced	
n	m	Z	$\omega\chi^2$	Z	$\omega\chi^2$
100	10	0.061	0.050	0.067	0.053
	20	0.053	0.043	0.053	0.043
	40	0.056	0.051	0.055	0.051
	80	0.057	0.053	0.058	0.053
500	10	0.061	0.050	0.066	0.053
	20	0.053	0.043	0.061	0.054
	40	0.056	0.052	0.060	0.054
	80	0.058	0.053	0.061	0.056

Web Figure 3. Power for the *multivariate* test under the quadratic (top) and trigonometric (bottom) deviations from the null. Shown are: Equally-spaced (black) and unequally-spaced (gray) timepoints for $n = 100$ (solid) and $n = 500$ (dashed) subjects. Note that the x-axis range differs between the quadratic and trigonometric deviations.

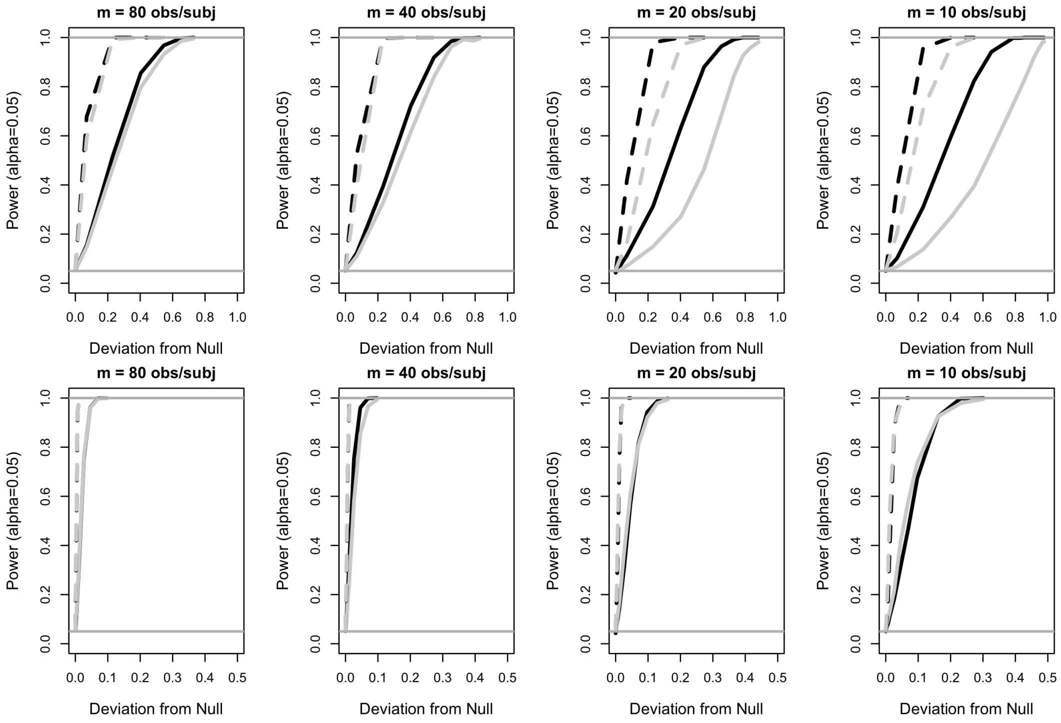

Received February 2018. *Revised* October 2018
